# Editorial: Streamlining drug approvals: addressing policy challenges in genitourinary oncology

**DOI:** 10.3389/fimmu.2025.1757428

**Published:** 2026-01-13

**Authors:** Guglielmo Mantica, Robert Hsu, Renate Pichler, David J. Benjamin

**Affiliations:** 1 Department of Surgical and Diagnostic Integrated Sciences (DISC), University of Genova, Genova, Italy; 2 Department of Internal Medicine, Division of Medical Oncology, University of Southern California/Norris Comprehensive Cancer Center, Los Angeles, CA, United States; 3 Department of Urology, Comprehensive Cancer Center Innsbruck, Medical University of Innsbruck, Innsbruck, Austria; 4 Hoag Family Cancer Institute, Newport Beach, CA, United States

**Keywords:** biomarkers, drug approval, drug policy, genitourinary oncology, GU

The field of genitourinary (GU) oncology is rapidly evolving. New biomarkers, molecules, immunotherapeutic agents, and targeted therapies are emerging faster than the approval and clinical use processes can keep up (1–3). While drug legislation and regulatory oversight remain essential to ensure safety and efficacy, they often create unintended barriers that delay access to potentially life-saving treatments. This tension between innovation and regulation, lies at the heart of this *Frontiers in Immunology* Research Topic, which explores the policy challenges that shape the approval and implementation of cancer drugs in GU malignancies. Over the past decade, many nations have sought to modernize their regulatory systems, introducing accelerated or conditional approval processes intended to bridge the gap between laboratory innovation and clinical availability. In some countries, fast-track pathways have succeeded in shortening timelines and expanding access, while in others they have introduced new uncertainties around evidence quality, cost-effectiveness, and long-term outcomes. Nowadays, GU oncology remain a field where rapid progress in systemic therapy has coincided with growing disparities in drug accessibility across different countries and healthcare systems. The articles gathered in this Research Topic collectively illustrate how policy, economics, and clinical science intersect in the management of GU cancers.

For example, Li et al. present an insightful cost-effectiveness analysis of *enfortumab vedotin plus pembrolizumab* as first-line therapy for metastatic urothelial carcinoma in the United States. Their findings underscore the tension between clinical benefit and financial sustainability, highlighting how even highly effective regimens can challenge the economic resilience of national healthcare systems. Similarly, the reviews by Liu et al. show the evolving landscape of first-line and maintenance therapies for advanced urothelial carcinoma, detailing how the regulatory environment influences the adoption of novel combinations and sequencing strategies. Finally, Inami’s work on the economic assessment of chemotherapy in gynecologic malignancies, though outside the traditional GU domain, offers a valuable methodological parallel, showing how cost-benefit modeling can guide rational drug use across oncology disciplines.

Taken together, these contributions reveal several recurring themes that transcend national boundaries. The first concerns the increasing reliance on surrogate endpoints to expedite drug approvals. A second theme is the economic dimension of drug policy, which remains unavoidable. The introduction of high-cost therapies, especially in immuno-oncology, poses a profound challenge for healthcare sustainability. Cost-effectiveness analyses, such as those featured in this Topic, are not merely academic exercises: they are increasingly central to reimbursement decisions and access negotiations. Transparent modeling and real-world data integration will be key to ensuring that accelerated approvals are accompanied by responsible pricing and equitable distribution.

Another central issue concerns international heterogeneity in prescribing authority and drug governance. In certain countries, oncologists hold exclusive prescribing rights for anti-tumor agents; in others, urologists and radiation oncologists may play an active role, at least for some agents. This lack of standardization fragments patient care and can hinder the efficient adoption of novel agents even after regulatory approval.

Furthermore, the studies included in this Research Topic also highlight the broader ethical dimension of regulatory policy. As we accelerate the path from discovery to patient care, we must not lose sight of transparency, equity, and public trust. Early access programs and conditional approvals can offer hope to patients with limited options, but they must be grounded in robust data and accompanied by commitments to ongoing evaluation. In this respect, policy innovation must move in tandem with scientific innovation: each is incomplete without the other. Ultimately, the future of GU oncology depends not only on the discovery of better drugs, but on the creation of smarter, more responsive systems to evaluate and deliver them.

## References

[1] Boehm BE York ME Petrovics G Kohaar I Chesnut GT . Biomarkers of aggressive prostate cancer at diagnosis. Int J Mol Sci. (2023) 24:2185. doi: 10.3390/ijms24032185, PMID: 36768533 PMC9916581

[2] Malinaric R Mantica G Lo Monaco L Mariano F Leonardi R Simonato A . The role of novel bladder cancer diagnostic and surveillance biomarkers-what should a urologist really know? Int J Environ Res Public Health. (2022) 19:9648. doi: 10.3390/ijerph19159648, PMID: 35955004 PMC9368399

[3] Yang T Luo W Yu J Zhang H Hu M Tian J . Bladder cancer immune-related markers: diagnosis, surveillance, and prognosis. Front Immunol. (2024) 15:1481296. doi: 10.3389/fimmu.2024.1481296, PMID: 39559360 PMC11570592

